# Impact of Quercetin Encapsulation with Added Phytosterols on Bilayer Membrane and Photothermal-Alteration of Novel Mixed Soy Lecithin-Based Liposome

**DOI:** 10.3390/nano10122432

**Published:** 2020-12-05

**Authors:** Sahar Pakbaten Toopkanloo, Tai Boon Tan, Faridah Abas, Fahad A. Alharthi, Imededdine Arbi Nehdi, Chin Ping Tan

**Affiliations:** 1 Department of Food Technology, Faculty of Food Science and Technology, Universiti Putra Malaysia, UPM, Serdang 43400, Malaysia; pakbaten.toopkanloo@gmail.com; 2 Department of Food Service and Management, Faculty of Food Science and Technology, Universiti Putra Malaysia, UPM, Serdang 43400, Malaysia; taiboon_tan@upm.edu.my; 3 Department of Food Science, Faculty of Food Science and Technology, Universiti Putra Malaysia, UPM, Serdang 43400, Malaysia; faridah_abas@upm.edu.my; 4 Chemistry Department, College of Science, King Saud University, P.O. Box 2455, Riyadh 11451, Saudi Arabia; fharthi@ksu.edu.sa (F.A.A.); inahdi@ksu.edu.sa (I.A.N.); 5 Chemistry Department, El Manar Preparatory Institute for Engineering Studies, Tunis El Manar University, P.O. Box 244, Tunis 2092, Tunisia; 6 Laboratory of Processing and Product Development, Institute of Plantation Studies, Universiti Putra Malaysia, UPM, Serdang 43400, Malaysia

**Keywords:** mixed soy lecithin, stability, phytosterols, membrane integrity, Fourier-transform infrared spectroscopy

## Abstract

This study used highly lipophilic agents with an aim to increase the oxidant inhibitory activity and enhance photothermal stability of a novel mixed soy lecithin (ML)-based liposome by changing the composition of formulation within the membrane. Specifically, the development and optimization of the liposome intended for improving Trolox equivalent antioxidant capacity (TEAC) value and %TEAC loss was carried out by incorporating a natural antioxidant, quercetin (QU). In this context, a focus was set on QU encapsulation in ML-based liposomes and the concentration-dependent solubility of QU was investigated and calculated as encapsulation efficiency (EE). To explore the combined effects of the incorporation of plant sterols on the integrity and entrapment capacity of mixed phospholipid vesicles, conjugation of two types of phytosterols (PSs), namely β-sitosterol (βS) and stigmasterol (ST), to mixed membranes at different ratios was also performed. The EE measurement revealed that QU could be efficiently encapsulated in the stable ML-based liposome using 0.15 and 0.1 g/100 mL of βS and ST, respectively. The aforementioned liposome complex exhibited a considerable TEAC (197.23%) and enhanced TEAC loss (30.81%) when exposed to ultraviolet (UV) light (280–320 nm) over a 6 h duration. It appeared that the presence and type of PSs affect the membrane-integration characteristics as well as photodamage transformation of the ML-based liposome. The association of QU with either βS or ST in the formulation was justified by their synergistic effects on the enhancement of the EE of liposomes. Parallel to this, it was demonstrated that synergistic PS effects could be in effect in the maintenance of membrane order of the ML-based liposome. The findings presented in this study provided useful information for the development and production of stable QU-loaded ML-based liposomes for food and nutraceutical applications and could serve as a potential mixed lipids-based delivery system in the disease management using antioxidant therapy.

## 1. Introduction

Liposomes can act as a suitable vesicle to enhance the solubility of hydrophobic bioactive compounds by encapsulating the compounds within the membrane bilayers. An appropriate composition of the lipid phase, along with formulation protocol and preparation techniques, will lead to the successful design of a liposomal system. Flavonoid quercetin (QU) (3,3′,4′,5,7-pentahydroxyflavone) is a semi-lipophilic molecule which is of great interest to researchers due to its great nutritional value. This compound has been shown, in vitro, to be a strong antioxidant and is one of the most powerful scavengers of reactive oxygen species, such as O^2−^, NO and ONOO- [1]. The free radical-scavenging effect of QU is based on its ability to donate proton. As reported by Boots and co-workers [2], QU can reduce inflammatory pain via the inhibition of oxidative stress and cytokine production. In addition, QU is able to interact and permeate lipid bilayer and such capacity is very important because there is a positive correlation between the ability to incorporate into membranes and antioxidant activity [3,4]. Once incorporated into membranes, QU changes the biophysical parameters of the membrane such as its fluidity, cooperativity and the temperature of phase transition. However, despite these beneficial properties, QU has a low absorption rate in the gastrointestinal tract, instability in physiological media and under the Biopharmaceutics Classification System, it is classified as a class IV compound (low solubility, low permeability) [5]. It is believed that low bioavailability of crystalline QU as a pure substance (as used in our study) is the result of its low solubility in the digestive tract [6]. The encapsulation method allowed overcoming the problems related to crystalline QU solubilization. Since sufficient QU solubility is only achieved in amphiphilic systems [7], one alternative to enhance QU bioavailability is by encapsulating it in amphiphilic nanocarriers, such as in mixed soy lecithin (ML)-based liposome systems. The fatty layer on the outskirts of the liposome confines and protects the enclosed compound material until the liposome travels to the site, adheres to the outer membrane of the target cell and delivers the payload [8]. The process avoids many of the transitional stages of a conventional delivery options. Therefore, development of novel nanovehicles that are capable of solubilizing QU to exert its bioactivity in inhibiting ultraviolet B-induced cutaneous oxidative stress and inflammation is of great significance. In accordance with this, correctly loading QU within the ML-based liposomes containing different membrane components is critically important for improvement of QU’s in vitro bio-accessibility because the lipid bilayers are affected by membrane components and exhibit a specific particle curvature and shape, which affect the in vitro digestion and dispersion capacities as well. In addition, the size and thickness as well as the entrapment efficacy and vesicular stability have been shown to be related to the composition of liposomal wall materials that can further affect its digestion behavior [9,10]. The use of soy lecithin as non-synthetic mixed phospholipids for producing liposomes does not raise any food legislation concerns and provides additional nutritional value owing to its high polyunsaturated fatty acids (PUFA) composition [11]. Nevertheless, the predominance of highly unsaturated fatty acids typically results in more permeable and less stable bilayers [12], and may eventually generate hydroperoxides and secondary oxidation products as a result of environmental stresses (mainly ultraviolet (UV) light), and whose accumulation in food/ nutraceutical systems could give rise to rancidity and a concomitant reduction in shelf life. Therefore, as a powerful antioxidant, QU is a good choice to be used as an efficient inhibitor against the degradation of ML-based liposomes as a result of light-induced oxidation.

It is of equal importance to produce stress-resistant liposomes via modulation of the lipid bilayers’ composition to protect the bioactivity of the encapsulated substances in the membrane, as well as to maintain the vesicles’ entirety. Like cholesterol, phytosterols (PSs) are also able to modulate a number of different membranes functions, such as acyl chain order [13], elasticity [14] and lateral organization [15]. Previous studies have investigated the addition of PSs to phospholipid vesicles, and demonstrated that their presence modifies the physical properties of the outer membrane [16]. However, despite the literatures, our understanding of the molecular basis of and specificity of the interactions with different PSs types and with mixed membrane phospholipids remain limited. In this regard, two types of PSs, namely β-sitosterol (βS) (C_29_H_50_O) and stigmasterol (ST) (C_29_H_48_O), were added in the liposomal formulations with the aim of improving the packing of the ML membranes. Moreover, incorporation of several biomaterials in an encapsulation system may enhance the bioactivity of individual components [17]. PSs have been shown to decrease the serum cholesterol levels and the ratio of the low-density lipoprotein (LDL) to high-density lipoprotein (HDL) bound cholesterol in serum [18]. In addition, PSs have been found to increase the oxidation stability of lipids [19].

Bilayer membranes are mosaic of areas maintained by the cytoskeleton network, whereby the lipid nature of the membranes is believed to play an important role in the formation of these areas. Squalene (C_30_H_50_), a natural isoprenoid compound, could be conjugated to phospholipids in the formation of liposomes with the intention of improving the membrane lipophilicity and consequently, the affinity towards the environment of lipid bilayers. As reported by Richens et al. [20], oily natural substances such as squalene can modify the membrane dipole potential. The adhesion capability of squalene could be affected by its lipophilic character and its tendency to integrate with the ML membrane bilayer. The “fit” between the two imperfect adjoining chains (unsaturated fatty acids) could adhere well after bringing squalene into lipid bilayers containing mono- and polyunsaturated lipid chains. In fact, aliphatic organic molecules have stronger interplays than aromatic compounds, because branches on a carbon chain will reduce the hydrophobic effect of that molecule, and a linear carbon chain can form the largest hydrophobic interaction producing steric hindrance by carbon branches. This dynamic is consistent with the findings of Ott et al. [21], who used squalene for dual delivery of hydrophilic and lipophilic actives. They declared that the steric hindrances of squalene could deliver postponed release functionality for nanostructured lipid carriers. Moreover, the addition of non-ionic surfactants such as Tween 80 was deemed to affect the hydrophilicity, particle size, fluidity and integrity of mixed liposomes [22]. The major difference between surfactant-incorporated liposomes, flexible liposomes and surfactant-free liposomes is the high and stress-dependent adaptability of such surfactant conjugated vesicles. This difference is demonstrated in the motive force given by the osmotic gradient between the outer and inner leaflets of the membrane of the liposomes. Edge activators with a high radius of curvature could definitely increase the fluidity and flexibility, and boost the deformability of the bilayers [23]. Apparently, such elastic membranes are suited for maintaining the integrity of the lamellar vesicles, breeding effective durability of the free radical scavenging system against UV-light-induced degeneration. Therefore, the use of various active ingredients for the fabrication of liposomes via an extrusion technology is expected to confer triple benefits; improved formation of stable liposomes for effective nutraceutical/ food-grade delivery applications, enhanced nutritional value through naturally-occurring bioactive compounds addition, and reduced photodegradation rate via the use of appropriate ratios of liposome ingredients.

Taking into account these notions, the aim of this study was to develop, optimize and characterize the liposomal formulation intended for antioxidant therapy by means of encapsulating QU with the addition of βS and ST. The main target of the research was to find the optimum formulation to produce QU-nanoliposomes with the highest encapsulation efficiency (EE), and highest stability (i.e., least degradation of the nanoliposomes against UV light as compared to other synthesized composites) using response surface methodology (RSM). Furthermore, this research aimed to provide a better understanding of QU physicochemical properties, stability and bioactivity in response to various factors and consequently focused on the physicochemical stability of the proposed ML nanoliposome system. The extrusion technique for nanosizing the liposomes was employed. The effect of adding the PSs (βS and ST) on photo quality, stability and potency of delivery properties of liposomes were investigated. Cured ML (instead of a pure specific phospholipid), a helper lipid (squalene) and a non-ionic surfactant (Tween 80) were used in the formation of the bilayers. This work also aimed to provide a structural understanding of the effects of the selected PSs on the stabilization, functionalization and encapsulation performances of the liposomes.

## 2. Materials and Methods

### 2.1. Materials

ML (composition as shown in Table 1) used in this research was sourced from Ncalai Tesque, Inc. (Kyoto, Japan). Squalene (99% purity; for synthesis) and polyoxyethylene sorbitan monooleates (Tween 80) were purchased from Merck Inc. (Darmstadt, Germany). Cholesterol (95%), QU (≥95%) and ST (≥95%) were supplied by Acros Organics Inc. (Branchburg, NJ, USA). βS (≥95%) was obtained from Amresco Inc. (Cleveland, OH, USA). All other chemicals and reagents, such as chloroform (CAS n. 67-66-3), and ethanol (CAS n. 64-17-5) (Sigma-Aldrich, Milan, Italy), used were of analytical grade. Deionized water was used in all experiments.

### 2.2. Liposome Preparation via the Extrusion Method

A pre-liposomal formulation containing 5.8% (*w*/*w*) of lipid was previously optimized in a separate study by varying the proportions of ML, cholesterol, Tween 80 and squalene containing 5% (*w*/*w*) ML, 0.3% (*w*/*w*) cholesterol, 0.75% (*w*/*w*) Tween 80 and 0.5% (*w*/*w*) squalene. The mass ratios of the pre-liposomal composition were selected based on our preliminary study and those reported by other studies [24,25]. The ML-based liposome was purified to formulate for the present study. In the next step, the optimal pre-liposome (OP_1_) was loaded with QU, βS and ST via a film-extrusion method based on established procedures [26]. Briefly, a thin lipid film, named as organic phase, consisting of the OP_1_ composition, with or without QU, βS and ST (see mass ratios in Table 2), was produced from a mixture of ethanol-chloroform (1:1 *v*/*v*) at a final volume of 10 mL. After the solvent removal by rotary evaporation (Rotary Evaporator, Ultra Lab, Delhi, India), the lipidic thin film was hydrated in phosphate buffered saline (PBS; pH 7.4, 0.05 M) and shaken using a magnetic stirrer (60 rpm, 1 h) above T_m_ (60 °C). The temperature above the T_m_ refers to liposome including the components mixture. The obtained liposomes were then homogenized using a bath sonicator (Loba Life, Mumbai, India) at 60 °C for 1 min to obtain a homogeneous suspension for the extrusion process. Finally, the lipid particles were extruded eight times through a 100 nm polycarbonate membrane (Millipore USA) filter by means of an Avanti Mini-Extruder (Avanti Polar Lipids, Inc., Alabaster, Alabama, USA) at above the T_m_ to achieve uniform particle size distribution. In this manuscript, the concentration of QU is always expressed as % *w*/*w* of the total formulation weight. Component ratios of lipid formulations are also always expressed in % *w*/*w*.

### 2.3. Particle Size, Polydispersity Index (PDI) and Particle Size Stability Measurement

The mean particle size (z-average), uniformity and PDI of the liposomes were assessed by dynamic light scattering (DLS) using a NanoZS90 instrument (Zetasizer Nano ZS, Malvern Instruments, Worcestershire, UK) at room temperature. All solutions were diluted 10-fold in PBS before measurement. The vesicular stability against UV light (280–320 nm) was analyzed to examine the impact of variable concentrations on physical properties of liposomes. All measurements were carried out 24 h after liposome preparation in order to avoid degradation of the phospholipids and to make sure that lipid vesicles were uniform in dispersion for particle size and PDI measurements. The liposomes were always stored in darkness and at 4 °C. Duplicate analyses were run for each sample.

The particle size value change rate caused by UV-light exposure given as a percentage was calculated using Equation (1):

(1) $$\begin{array}{l} \mathrm{Value}\;\mathrm{change}\;\mathrm{rate}\;\left(\%\right)\\ \;=\frac{\left[\mathrm{UV}\;\mathrm{treated}\;\mathrm{particle}\;\mathrm{size}\;\left(\mathrm{nm}\right)-\mathrm{Initial}\;\mathrm{particle}\;\mathrm{size}\;\left(\mathrm{nm}\right)\right]\times 100}{\mathrm{Initial}\;\mathrm{particle}\;\mathrm{size}\;\left(\mathrm{nm}\right)} \end{array}$$

where the value change rate represents the percentage increase of the particle size of liposomes after one time submission to UV-light irradiation.

### 2.4. Zeta Potential (ZP)

The stability in the term of surface charge was further investigated by determining ZP 24 h after liposome preparation using the Malvern Zetasizer Nano series (Zetasizer Nano ZS; Malvern Instruments Ltd., Worcestershire, UK). The liposomal preparations were gently dispersed in PBS (10 fold) at room temperature to produce a yellow suspension prior to the measurements. All measurements were performed in triplicates.

### 2.5. Encapsulation Efficiency of QU (EE_QU_)

Standard curves were prepared using stock solution of 1 mM QU and dimethyl sulfoxide (DMSO) as a blank. The calibration curves were linear (R^2^ > 0.996) against the QU concentration (1–10 µg mL^−1^).

Entrapment efficiency of QU-loaded liposome was determined by assessing the difference between the total amount of QU and the amount of free QU present in the liposome. The amount of QU encapsulated in the QU-ML-based liposomes was identified by an indirect method via centrifugation technique using a centrifuge (Model: 3740, KUBOTA MFG. CORP, Tokyo, Japan) at 5000 rpm for 15 min at 4 °C. The experiment of QU quantification was carried out at 370 nm [27]. The concentration of QU was measured via UV/V is spectrophotometry (Agilent Technologies, Basel, Switzerland) at 370 nm. The *EE_QU_* was then calculated according to Equation (2):

(2) $$\%EE_{QU}=\frac{\left(W_{total}-W_{free}\right)\times 100}{W_{total}}$$

where *W_total_* is the total *QU* weight in liposomes suspension; *W_free_* is the weight of free QU.

### 2.6. Assessment of DPPH (2, 2-diphenyl-1-picrylhydrazyl) Scavenging Rate, and DPPH Scavenging Rate of Loss against UV Light

The percentage of antioxidant activity was evaluated via the DPPH method [28]. Sample solutions were allowed to react for 30 min with DPPH (100 µM) in the dark, subsequently the decrease in absorbance of the liposomal samples was measured at 517 nm using UV–Vis spectroscopy (Agilent Technologies, Basel, Switzerland). The control mixture was prepared by replacing the sample with 50 µL of ethanol. Lower absorbance of the reaction mixture indicated higher radical-scavenging activity. The percentage inhibition of radical scavenging activity was calculated by Equation (3)

(3) $$\%\mathrm{Antioxidant}\;\mathrm{activity}=\frac{Ac-As\times 100}{Ac}$$

where *As* and *Ac* represents the absorbance of the sample and the control, respectively.

The percentage of antioxidant activity was expressed in Trolox equivalent antioxidant capacity (*TEAC*). Often, Trolox is used as a reference compound and the capacity is expressed as Trolox equivalent [29].

Trolox (0.250 g) was dissolved in 50 mL of 75 mM phosphate buffer (pH 7.4) to make a 0.02 M stock solution for the preparation of working solutions of Trolox (0, 25, 50, 100, 150 and 200 µM) [30]. The *TEAC* corresponds to the Trolox concentration (µM). The *TEAC* was determined using the following Equation:

(4) TEAC (µM); y=0.4178x−5.9073

Equation (4) was obtained from a linear least squares fit (R^2^ = 0.9809, n = 5) to a plot of Trolox versus %antioxidant activity. Of this, “*x*” refers to *TEAC* value and “*y*” refers to %antioxidant activity value obtained from Equation (3).

The loss/degradation of *TEAC* (%) as a result of photodegradation (280–320 nm, 6 h) was then obtained as follows (Equation (5)):

(5) $$TEAC\;\mathrm{loss}/\mathrm{degradation}\;\mathrm{rate}\;(\%)=\frac{\left({TEAC}_{b}-{TEAC}_{a}\right)\times 100}{{TEAC}_{b}}$$

where *TEAC_b_* and *TEAC_a_* are the percentage of *TEAC* before and after UV-light irradiation, respectively.

### 2.7. Fourier-Transform Infrared Spectroscopy (FTIR) Assay

Infrared absorption spectra of the three added compounds (QU alone, βS alone and ST alone), free liposomes and liposomes with QU, βS and ST addition were recorded using a Fourier-transform infrared absorption spectrometer (FTIR-8400S, Shimadzu, Japan). Absorption spectra at a resolution of one data point every 0.6 cm^−1^ were obtained in the region between 4000 and 750 cm^−1^ using a clean crystal as the background. All experiments were performed at 20 °C.

### 2.8. Thermal Stability

The optimal liposomes (OP_1_ and OP_2_) were transferred into boiling tubes with screw caps and immersed in water bath set at different temperatures (30–110 °C) for 30 min, then immediately cooled to room temperature under running water. Samples were then stored overnight at room temperature prior to particle size and uniformity measurements.

### 2.9. Transmission Electron Microscopy (TEM)

Liposomes with QU, βS and ST and the control liposome were visualized via negative stain electron microscopy. A drop of the liposome suspension (~0.1 mg/mL) was deposited onto on a carbon film-coated copper grid. After 60 s, excess solution was removed by tapping the edge of grid with filter paper. A drop of 1% uranyl acetate solution was then applied to the same grid for 60 s. The grid was again tapped dry and further dried in the desiccator overnight. Images were taken on a transmission electron microscope (Hitachi H-7100, Tokyo, Japan).

### 2.10. Experimental Design and Statistical Analysis

RSM with a three-factor, two-level Box-Behnken Design (BBD) was used to optimize the composition concentrations of the liposomal formulations. Three independent factors were studied, specifically QU dosage (% *w*/*w*), βS dosage (% *w*/*w*) and ST dosage (% *w*/*w*), at 3 different levels for each (Table 2). Preliminary experiments were carried out to obtain the ranges of the studied parameters. The response measured were the liposomal particle size (Y_1_), ZP (Y_2_), EE (Y_3_), TEAC (Y_4_), liposomal particle size value change rate (Y_5_) and percentage loss of TEAC (Y_6_), as shown in Table 3 and Table 4. The experimental plan was designed and the results obtained were analyzed using Minitab 16 (Minitab Inc., State College, PA, USA). Each term of the model was tested statistically, and *F*-ratio significance was confirmed at a *p*-value of 0.05, as determined by Tukey’s test. Verification of model validity was confirmed by comparing the experimental data with the predicted results from the optimized model.

## 3. Results and Discussion

### 3.1. Particle Size, PDI and Particle Size Stability

The ability of the three added substances to interact with the mixed phospholipids could be confirmed via particle size and PDI examinations. According to DLS results (Table 3), it is revealed that all the liposomal systems prepared were nanosized, ranging from 158 to 184 nm. The PDI was low for all the formulations (<0.2) (Table 4). A significant (*p* < 0.05) particle growth for βS and ST-liposomes in the quadratic range was observed (Table 5). Furthermore, their interaction effect was shown to have a significant impact on the particle size and PDI. This is in line with literature reports of larger size and lower absolute ZP for plant sterol-containing liposomes [31,32]. As detailed in Table 6, addition of βS in linear range led to formulations with lower PDI (*p* < 0.05). However, the addition of QU appeared to have no significant impact (*p* > 0.05) on the particle size and PDI.

These results suggest that liposome membranes displayed irregular thickness and increased lipophilicity after the inclusion of additional membrane components (βS and ST) and the subsequent alteration in the assembly of the vesicles. The term “thickness” here hints at an increase in the diameter of the particles and thus the unsteady membrane as a result of adding the bigger amount of the PSs. The attachment efficiency and lipophilic character of PSs could be influenced by their excessive amount, leading to weak merging with the ML membrane bilayer. The results seemed to indicate that highly lipophilic agents induced membrane structural changes and vesicle stability of ML-based liposomes. It is, therefore tempting to conjecture that the sterol tilt modulus parameter provides an important energy-based measure of the condensing effect of sterols on lipids, and with that, complements structural measures such as area per lipid, membrane thickness or lipid tail order parameter that are routinely used to describe cholesterol condensation. In general, the creation of unbalanced amphiphilic structures leads to fluctuation in membrane curvature and increased asymmetry in the geometry of the self-assembly. This is also likely to impose changes on the fluidity and packing density of the polar-nonpolar interface of the phospholipid head group area at the surface of the liposomes. A major consequence of these changes is considerable leakage of the entrapped compounds that have been attached to the bilayers, and size growth. Moreover, a large amount of variation have been reported as the effect of sterols on the thermotropic phase behavior and organization of bilayers composed of different membrane lipids containing fatty acids with different chain lengths and a number of unsaturated bonds [33]. It is also believed that the dissimilar lipid packing is more likely to affect the orientation and degree of penetration of different types of lipophilic substances (here, βS and ST) into the hydrophobic core of the lipid bilayer and as a result, form unsteady lipid vesicles [34]. Therefore, it is important to keep in mind that the lipid ordering of bilayers using the ML was as critical as using the different types of PSs in the liposomal formulations. Remarkably, treatment F4 containing the highest total PSs mixture displayed the largest particle size (184 nm). In this context, the bigger amount of the PSs combination could make gel-phase phospholipid bilayers too stiff to be extruded easily to produce particles with fairly uniform and small size [34,35].

**Figure 1:**
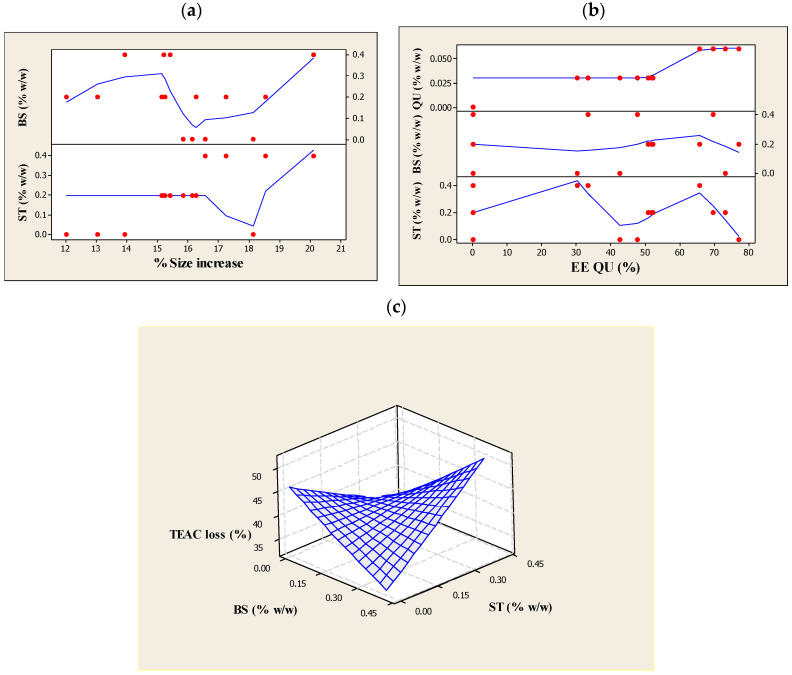
The effects of (**a**) β-sitosterol (βS) and stigmasterol (ST) concentrations on size increase (%); (**b**) quercetin (QU), β-sitosterol (βS) and stigmasterol (ST) concentrations on the quercetin (QU) encapsulation efficiency (%EE_QU_); and (**c**) β-sitosterol (βS) and stigmasterol (ST) concentrations on Trolox equivalent antioxidant capacity (TEAC) loss (%) after ultraviolet (UV) light exposure (280–320 nm, 6 h) of the liposomes prepared using different compositions.

The particle sizes for all formulations were evaluated before and after UV-light treatment (290–360 nm, for 6 h) to investigate the physical stability of the produced liposomes. Table 3 summarizes the changes in particle diameters of liposomes in response to UV light. The data prove that UV-light radiation had a destabilizing effect since the liposome size showed an increase. This may be due to the alterations in the membrane structure upon hydrolysis and oxidation of the ML [36]. However, as compared to pre-liposomal compositions, they exhibited a lower percentage increase, and this indicates the effective role of QU and PSs in the formulation (Figure 1a). The analysis revealed that the value change rate of particle size was positively affected (*p* < 0.05) by the βS addition, but varied only slightly (*p* > 0.05) with the addition of QU and ST in the formulations (Table 6).

Generally, it is considered that oxidization and crosslinking are two of the major mechanisms of photoreaction occurring in the unsaturated bonds, which promote the formation of pores in the lipid bilayers and the concomitant membrane leakage. The addition of PSs could modulate the bilayer thickness and elasticity by arranging themselves in parallel rows between the layers [14]. Parallel refers to the position occupied by sterols within the bilayer membrane. As stated by Murari et al. [37], sterols are aligned parallel to the bilayer normal. According to Dufourc [38], PSs are able to strongly reinforce membrane cohesion. Moreover, it was pointed out by Gao et al. [13] that the interaction of the sterol alkyl side-chains and lipid acyl chains can play a role in stabilizing bilayers due to the minimization of the hydrocarbon chain mismatch. Different types of sterol molecules (βS and ST in this case) induce different membrane formation characteristics, leading to differences in molecular packing of the mixed phospholipids (mismatch). Therefore, PSs can protect capsule membrane of photo-labile lipids against photodecomposition upon UV-light irradiation and thus maintain the assembly of lipid vesicles. However, it was also reported that the ST’s tendency to induce lipid bilayers is deemed to be limited [39]. As the deuterium nuclear magnetic resonance (^2^H NMR) experiments carried out by Schuler and co-workers clarified, sitosterol is highly capable of ordering PC acyl chains to reduce water permeability while ST is the PSs that showed a less hydrophobic matching, which refers to less equal/accordant hydrophobic interactions within the bilayer or equal bonds between the alkyl chain of phospholipids and a hydrophobic segment of sterols) for phospholipid chains [40]. The findings above suggest that the addition of βS in the membrane (instead of ST) resulted in a better packing to feasibly counteract the photon energy. Indeed, sterol molecules in disordered environments, as found near polyunsaturated lipids in stressful conditions like UV light, can more easily orient themselves perpendicular to the bilayer normal and reside close to the membrane center, where they enjoy greater orientational freedom compared to the low orientational entropy “standing up” configuration [41].

Table 6 shows a major (*p* > 0.05) increase in the %size increase values during UV irradiation through engaging βS and ST collaboratively (X_2_X_3_) in the liposome formulation, which is in agreement with the results of the initial size (see Table 5). As pointed out previously, this growth might be due to the differences in the packing of the mixed phospholipids resulting from the distinct induction of different types of PSs molecules (here, βS and ST) on the membrane-shaping properties. In other words, the ability of the sterol molecules to fit into the free space between various phospholipid molecules in the bilayer core can be essentially limited with respect to one another [42]. Thereupon, by presenting the different PSs fragments and concentrations in the lipid membranes, the hydrophobic matching between the alkyl chain of phospholipids and a hydrophobic segment of sterols might be reduced. The subsequent increase in the mismatches of lipid networks might expose some potential interferences to the liposome membranes upon UV-light excitation; first, it makes the lipid membranes susceptible to a substantial fragmentation and increased membrane permeability; second, creates space between the layers and increases the possibility that the terminal lipid methyl groups will be exposed at the aqueous interface and are more likely to come in contact with water and then undergo hydrolysis.

Finally, another possible reason that resulted in the liposomes having a significant large size was the presence of free unsaturated fatty acids in the liposome formulations, which might have affected the membrane intactness against UV light. The PUFA chains with their multitude of rapidly changing conformations could push away the rigid steroid moiety as PUFA-containing phospholipids have poor affinity for sterol due to the highly disordered PUFA chains. Similar findings were reported by [43].

### 3.2. ZP of the Produced Liposomes

ZP is also an important parameter that allows the prediction of the liposomes’ physical stability. All liposomal formulations prepared in this study presented a negative charge, varied from −23.28 to −42.82 mV. As detailed in Table 5, the presence of QU in quadratic range was a positive significant (*p* < 0.05) contributor to ZP values. It appeared that QU presents a high affinity for liposome membranes owing to its planar configuration. This flavonoid possesses an aromatic part which has affinity for the lipid component-hydrophobic environment. Concurrently, the presence of five hydroxyl groups suggests a propensity for a polar environment [44]. As such, we believed that the increased amount of QU that was available in the liposomal dispersion would link the two phases (organic and aqueous phases) together, thus providing a homogeneous (uniform appearance and texture) colloidal dispersions. From another perspective, it is thought that QU hydroxyl groups were probably not totally inserted into the lipid bilayers. Therefore, QU could act as a semi lipophilic molecule and develop molecular interactions with phospholipids in the water subphase [45]. Based on this consideration, it seems feasible to suggest that such molecular interactions among the organic and water phases could form an ordered close packing that strengthens the interfacial layer and was likely to increase the colloidal stability of the liposomal dispersions.

Interaction terms of QU with either βS or ST (X_1_X_2_ and X_1_X_3_) (see Table 2) showed considerable (*p* < 0.05) beneficial effects on the ZP values (Table 5). The results also revealed that ZP values were greatly (*p* < 0.05) reduced by the contribution from pair interaction of βS and ST (Table 5). Without QU, liposomes prepared with more βS and/or ST exerted an adverse effect on the ZP range, although this effect was not significant. Based on these findings, the association of QU with either the βS or ST in the formulation was justified by their synergistic effects on the enhancement of the surface charge of liposomes.

Technically, higher ZP values (either positive or negative) serve to stabilize particle suspensions. In our liposomal dispersion, it might be that the encapsulation of QU improved the physical properties of the mixed membranes of lecithin (containing unsaturated fatty acids), appertaining to ordering of the acyl chains and subsequent reduction of membranes distortion [46]. By this, it is more likely that the PSs molecules could organize themselves successively between the layers. In addition to this, the presence of one of the PSs could be beneficial against the fluidity of the membrane because PSs are predominantly lipophilic in character and are able to fully fit in the hydrophobic compartment of the bilayers through steric fitting and hydrophobic interactions. So the interplay of the PSs and mixed phospholipids would primarily persuade the QU molecules to strongly align along the bilayer, and with that, they became more strongly engaged in interactions with neighboring lipids and this increased the vesicle-forming stability. Moreover, such interactions with the mixed phospholipids could contribute to restricting the movements of fatty acyl chains in the membrane area and thereby stable fluid bilayers could be obtained. This statement agrees well with the results of Webb and co-workers who proposed that once the sterol molecules participate in membrane formation, they decrease the hydration and the effective size of the polar head group of phospholipids and bring more compressible lateral packing of acyl chains inside the bilayers [47]. Hence, it would be more difficult for sterol-contained liposomes to break and merge together.

Not only that, it is thought that PSs can affect the liposome surface charge since they can improve the bilayer properties. There may be a few reasons to this. Firstly, the position occupied by sterols (here, βS or ST) in the membrane bilayer can slightly ionize fatty acids from their polar part and result in additional negative charge. This ionization helps to increase the electrostatic repulsion among vesicles and avoid aggregation [48]. Secondly, non-ionic surfactants/sterols interactions and phospholipids/sterols interactions via hydrogen bonds can promote lipid mixing and reinforce the packing of the bilayers owing to increased interactions [49]. Simply put, the inclusion of hydrogen bonds strengthened the hydrophobic interaction with fatty acyl chains and suppressed the electrical double layer electrostatic interactions, which further inhibited membrane deformation and vesicles aggregation. Hence, a higher ratio of QU penetration into the phospholipid bilayer could modify the ZP.

### 3.3. Encapsulation Efficiency of QU (EE_QU_)

The EE_QU_ (Table 4) in liposomes varies from 30.22 to 77.13% (in formulations loaded with QU), and it was clearly affected by the QU concentration, as it increased significantly (*p* < 0.05) in the quadratic term of QU. This is reasonable due to the fact that the QU molecules could dissolve well in the unsaturated fatty acid moieties of the ML. It could be that the QU molecules participated in the mixed interdigitated bilayer with good affinity for unsaturated phospholipids, which increased the total amount of QU encapsulated. It is worth noting that the addition of lipophilic agents (squalene in the midplane of the bilayer) further could enhance interdigitation in ML bilayer. After adding lipophilic agents an interdigitated gel phase in fully hydrated phospholipids bilayers were formed (change from gel phase (L_β_) to gel interdigitated (L_β1_)) [50]. In addition, Tween 80 could sterically shield the lytic effect of phospholipids in the interface most likely by partitioning into the interfacial region and reducing the exposure of the terminal methyl groups to aqueous phase [51]. Another reason could be because of the use of flexible membrane, which is believed to have a beneficial effect on the liposomal entrapment [52]. Liposomes composed of the mobile hydrophilic-type surfactants such as Tween 80 can form an elastic membrane [44], whereupon changes in the fluidity and packing density of the polar-nonpolar interface occur and can further improve the QU entrapment efficacy. Liu and co-workers also reported a higher EE for QU in liposomes containing Tween 80 [44]. Beyond this, the polyunsaturated lipid, squalene, in the midplane of the lipid bilayer might increase acyl chain dynamics and therefore create a more attractive environment for the encapsulation of QU. Considering the results and the above discussions, the use of 0.06% (*w*/*w*) of QU resulted in the liposomes having the desired particle size and enhanced particle size stability with a higher EE.

The linear terms of βS and ST showed a major increase (*p* < 0.05) in terms of EE_QU_, which was influenced more by βS (see Figure 1b). The highest EE_QU_ could reach up to nearly 77% in the presence of βS in the formulation. This may be attributed to the improvement in the structural features (e.g., stiffness and ordering) of bilayers. As indicated by Schuler and co-workers, the intercalation of PSs into phospholipid vesicles modifies the physical qualities of the outer membrane, specifically the molecular packing of the phospholipid bilayers [16]. This affects the delivery properties of the vesicles formed; in particular, an increase in the EE of the vesicles. It is also stated by Chen and co-workers [53] that sterols can fill the gaps within bilayers and minimize leakage. Given these findings, the βS and ST-containing liposomes might enhance the lipophilic character of QU and assure a better interaction of QU with the liposome membranes.

For quadratic terms of βS and ST, a significant loss of EE_QU_ occurred (Figure 1b) suggesting that a change in membrane packing order resulted in adverse effect on the entrapment capacity. These findings are also supported by the measurements of the particle size. As can be seen from Table 5, the effect of a higher concentration of ST was much more destructive than βS on encapsulating capability of the ML liposomes. Structurally, ST has branched and unsaturated chains at the C17 position. This bulky alkyl tail chain yielded a less tight phospholipids chain packing [49] and perhaps some of the components segregated in localized discrete areas, creating a lateral heterogeneity. Thus, this could have a detrimental impact on the process of the membrane forming with QU. Additionally, it was confirmed with ^2^H NMR study [40] that ST caused the phospholipid chains to result in a less ordered and tilted gel phase that could lead to phase separation at higher concentrations. Similarly, in our liposomal systems, the presence of a high concentration of ST might result in a disordered lipid lattice with many flaws. Bringing it all together, it is important to make clear, at least in general terms, how βS could positively affect QU encapsulation, while considerable reduction of EE_QU_ was observed in the presence of ST? These may simply be due to the differences in the packing of the phospholipids resulting from the use of different PSs molecules in the mixed membrane of lecithin because the two PSs have different behaviors and as a result, they influenced the liposome entrapment. Sitosterol could increase the segmental order parameters more than ST; the difference being even higher when considering the chain positions near the bilayer center [54]. Even more, the difference between the former and the latter by the extra double bond in the C22=C23 position of the latter reflects the distinct roles in the regulation of the membrane properties [40]. Based on the results achieved by the force area curves experiment that was done on the mixtures of lecithin with the plant sterols, ergosterol and ST [55], it was found that a smaller condensation effect and a less effective interaction in monomolecular layers were due to the double bond at C-22, 23. These findings suggest a correlation between the plant sterol-inducing membrane ordering and the increase of the lipid core thickness. Hence, it is not surprising that the lipids in the ST-containing membrane were significantly less ordered than those in the βS-containing membrane. In general, EE% measurement indicated that QU could be encapsulated in stable liposomes using 0.15 and 0.1 g/100 mL βS and ST concentrations, respectively, with 0.06 g/100 mL QU as being highly effective, which means the two PSs were able to increase the EE_QU_ when present at the linear range. Nevertheless, βS was more efficient than ST at enhancing the EE_QU_.

### 3.4. Assessment of TEAC, and the Percentage Loss of TEAC against UV Light

The scavenging effect of the liposomes on DPPH radical was evaluated. The TEAC values significantly (*p* < 0.05) improved through the addition of QU in the quadratic range, and with using either βS or ST in the linear range (Table 5). The results confirmed that the inclusion of a favorable quantity of a flavonoid and a plant sterol (βS or ST) in the ML-based liposomes was advantageous for scavenging DPPH. In addition to this, the combination of phospholipids and a plant sterol led to the formation of a closely packed mixed film; this conferred steadiness to the lipid network and improved stability after preparation, which is attributed to the steric stabilization of the PSs [31]. Notably, a high value of TEAC loss after UV exposure was reported in the liposome without any PSs (see Table 4).

However, further analysis showed that the TEAC values experienced a dramatic (*p* < 0.05) decrease from using βS and ST in the quadratic term (Table 5). It is suggested that a crucial interfering force had ensued in the lipid chain order, and this was responsible for the deteriorating DPPH scavenging process in the liposomes. This might have occurred because the amphiphilic balance perturbed the geometry of the self-assembly. Firstly, the solute (QU, βS and ST are the solutes here) might prompt an elastic perturbation of the lipid bilayer. This elastic perturbation is an outcome of the solute’s shape and size, which the lipid bilayer tends to adapt because of the strong hydrophobic coupling between the solute and the membrane. Secondly, the presence of a rigid solute reduced the conformational freedom of the neighboring lipid chains. Basically, because lipid chains cannot penetrate into the rigid solute, the number of accessible chain conformations and orientations is smaller in the vicinity of the solute than far away from it. From another perspective, in the system with increase amounts of βS and ST, the amphiphilic balance of free fatty acid would cause a considerable proportion of the present PSs to diffuse out of the bilayer, and the lamellar structure exposed to some perturbation. The produced mixed phospholipids liposomal system in this study was a mixture of very different lipid components (various phospholipid model membrane systems), which may affect the characteristics of the whole system and its incorporated compounds. Moreover, the differences here may also be explained by the fact that we utilized various saturated and unsaturated phospholipids. Since sterols are known to be less soluble in highly unsaturated phospholipid bilayers [56] and such effects may be exaggerated in the case of using other lipophilic compounds, mixed phospholipids unequal affinity for different sterols provokes the formation of domains [57]. As it is stated previously by the authors [58], an increase in alkyl chain volume was extremely effective in sterol condensing capacity. However, additional methyl groups in sterols’ ring system markedly counteract this rigidifying effect. They have also mentioned that the sterols with the bulkiest unsaturated side chains or sterol nuclei (ST, βS and lanosterol) induced the smallest order parameter increase of the fluid bilayer at high sterol concentrations (>30 mol %), and hence became less potent rigidifiers at high sterol levels.

As depicted in Table 3, the changes in the TEAC values after UV irradiation procedure for 6 h was monitored in terms of assessing the loss of DPPH scavenging rate against UV light. We observed that the value of TEAC loss was significantly (*p* < 0.05) reduced when an increased amount of QU was applied in the formulation. This was within expectation because the QU could act as a potent radical-scavenger in the produced liposomal systems, though it is important to note that 0.06% (*w*/*w*) was the efficient amount of QU that showed sufficient solubility in the mixed amphiphilic systems (see also %EE results). Once the photodecomposition (i.e., separation due to the energy of light) occurs, the energy from the photon breaks the molecule apart. It becomes two separate oxygen atoms. The successful capture of the QU molecules inside nanoconfinements afforded by the ML-based vesicles could explain an increased effectiveness of the liposomes against UV-light damage. Specifically, flavonoids skeleton contains a planar chromophore with an additional carboxyl group for protonation and can effectively intercalate into the planar membrane of phospholipids. Likewise, the QU molecules with aromatic side groups and double bonds could potentially absorb UV energy. This observation is in agreement with previous reports [59,60]. Additionally, it is proved that the QU chromophore that embeds into the liposomes is located in the head group area at the surface rather than in the hydrophobic core of the lipid bilayer [61]. Therefore, most of the light could be absorbed by the QU readily at the surface, protecting the molecules in the inner area of the bilayers and thus preventing physical damage of the membrane and resulting in a more efficient DPPH scavenging activity. Most of the light will be absorbed close to the sample surface if a solution contains the compound substance in high concentration. The strongest antioxidant capacity of QU with liposome particles suggests that this kind of mixed lipid particles can provide a better microenvironment for the 3′ and 4′ hydroxyl groups in ring B of QU, which are not participating in the formation of hydrogen bonds, and easily donate H+ to reduce DPPH into its nonradical form. These statements are explained in terms of the binding of QU to the hydrophobic pockets of lipid particles mainly through the hydrophobic force together with the hydrogen bonding. The hydrophobic binding of the aryl groups of QU to the hydrophobic pockets of liposome particles results in an increase of fluorescence intensity and anisotropy of QU [62], which was caused by the absorption ability of QU chromophore against UV light [63]. As reported by Bai et al. (2019) [64] carboxymethyl chitosan-QU composite films showed much better UV-light barrier ability than carboxymethyl chitosan film, which was caused by the absorption ability of QU chromophore against UV light.

Our results also showed that when conjugated with either βS or ST, a significant (*p* < 0.05) improvement of the TEAC loss was observed in the liposomes. Nonetheless, their interaction influence was significantly less potent in this context, leading to the most crucial increase in the percentage loss of TEAC values (see Table 6 and Figure 1c). The reported improvement suggested that both of the βS and ST-enriched membranes could create a favorable environment for QU.

On the other hand, the detrimental impact of βS and ST interactions in the inhibition stability could be ascribed to (i) severe photochemical reactions not only from intersterol interaction, but also from free radicals and sterols reaction and then bringing about the oxidation of the steryl moiety [65,66], (ii) PSs oxidation as a result of autoxidation reactions and formation of PSs oxidation products [67], (iii) changes in the membrane composition after differing in a combined intake of the βS and ST, which caused variations to hydrophobic characteristics, and eventually introducing a fluid character in these bilayers. Accordingly, when a membrane undergoes bending deformations (in response to stress such as UV light), the sterol molecules are forced to rearrange in the membrane and on average, assume a more splayed configuration with respect to one another [68]. Such membrane disturbances might be able to pivotally diminish the antioxidant potency of the QU in regards to the ability to penetrate the membrane. Nevertheless, applying βS or ST in the linear terms in the membrane of liposomes would result in a decrease in the volume of the hydrophobic phase, modifying the ability of the antioxidant to interact with and penetrate the lipid bilayer. The antioxidant activity of flavonoids appeared to be governed not only by their structural features but also by their location and degree of penetration into the membrane.

### 3.5. Structural Analysis by FTIR Spectroscopy

**Figure 2:**
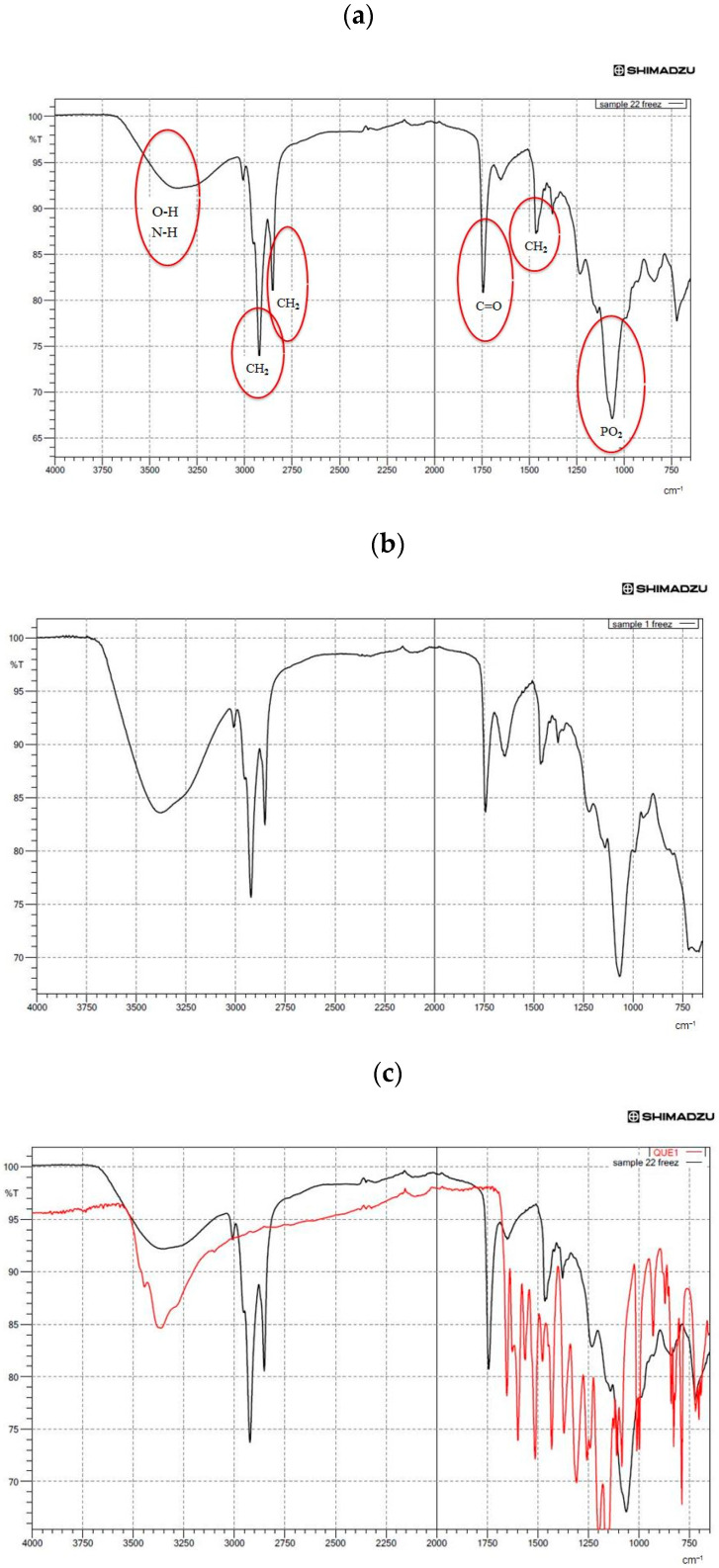

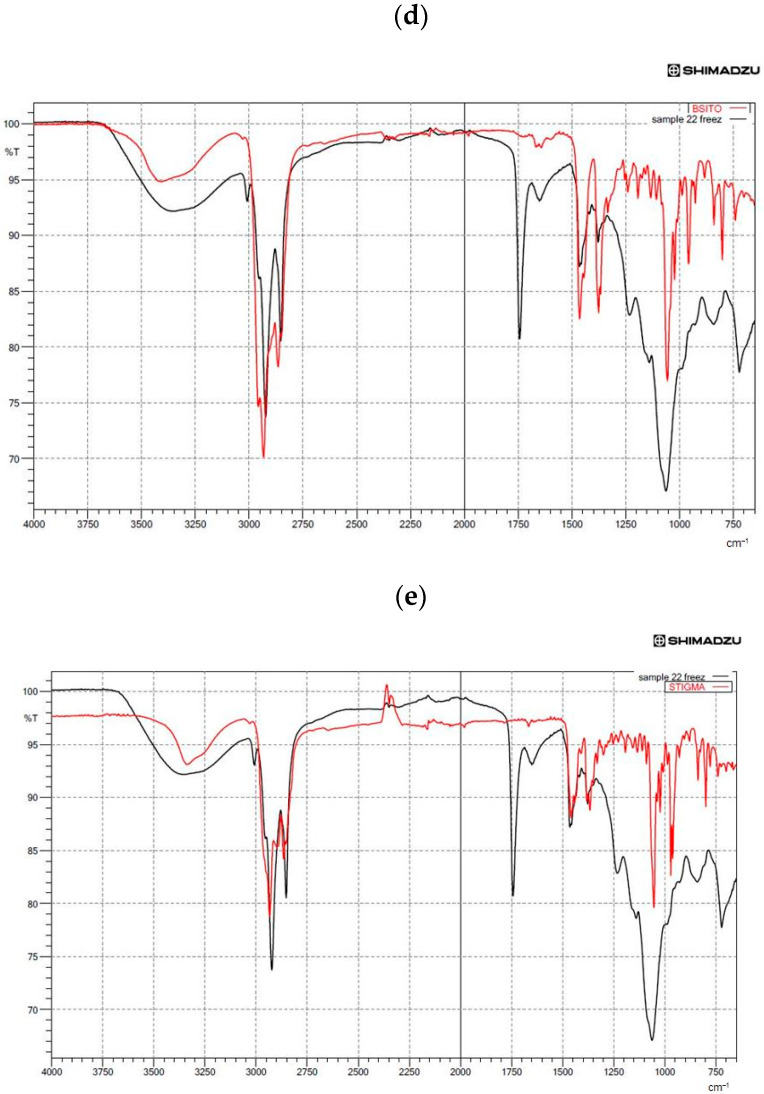
Fourier-transform infrared spectroscopy (FTIR) spectra showing (**a**) the spectra of quercetin (QU)-loaded mixed soy lecithin (ML)-based liposome stabilized by β-sitosterol (βS) and stigmasterol (ST), (**b**) the spectra of quercetin (QU)-loaded mixed soy lecithin (ML)-based liposome without β-sitosterol (βS) and stigmasterol (ST), (**c**) the overlap spectra of pure quercetin (QU) (red) and quercetin (QU)-loaded mixed soy lecithin (ML)-based liposome stabilized by β-sitosterol (βS) and stigmasterol (ST) (black), (**d**) the overlap spectra of pure β-sitosterol (βS) (red) and quercetin (QU)-loaded mixed soy lecithin (ML)-based liposome stabilized by β-sitosterol (βS) and stigmasterol (ST) (black) and (**e**) the overlap spectra of pure stigmasterol (ST) (red) and quercetin (QU)-loaded mixed soy lecithin (ML)-based liposome stabilized by β-sitosterol (βS) and stigmasterol (ST) (black).

FTIR spectroscopy experiments were carried out to examine the possible reactions between QU and the liposome, and the inclusion of the PSs and the liposome. The following spectral regions were revealed: amino +NH, hydroxyl–OH asymmetric stretching vibration (1620–1660 cm^−1^), phosphate asymmetric stretching vibration PO_2_^‒^ (1220–1260 cm^−1^), carbonyl stretching mode C=O (1680–1800 cm^−1^) and symmetric CH_2_ stretching vibration (2850–2855 cm^−1^) (see Figure 2a,b). As illustrated in Figure 2c, characteristic bands for pure QU at 1381 cm^−1^ (C-OH) and 1264 cm^−1^ (C-O-C) were obtained. These bands disappeared in the spectrum of QU-liposome, which indicated that QU could be well-loaded in the lipid bilayers, probably by hydrophobic interactions or hydrogen bonds [44]. Peaks at around 1010 and 1600 cm^−1^ in the Q-NPs spectra were associated with aromatic bending and stretching. The ν(OH) band was located in the 3600–2000 cm^−1^ vibrational region of the IR spectrum. The shape and position of this band strongly depended on the strength of a hydrogen bond formed by hydroxyl groups. As shown in Figure 2c, the band of the stretching vibration of OH groups in pure QU was sharp. The shift to lower wavenumber region accompanied by broadening of this ν(OH) band was characteristic of hydrogen-bonded OH groups. This shift increases proportionally to the increase of the strength of the hydrogen bond [69].

Figure 2c,d present the IR absorption spectra in the region characteristic of the symmetric C–H stretching vibrations of methylene groups (2800–2900 cm^−1^) of lipid alkyl chains that form a hydrophobic core of the lipid bilayer. As can be seen from the analysis of the position of this band (Figure 2d), overlapping with the CH_2_ groups of the liposome and βS was found. On the contrary, our results demonstrated a little overlap of its CH_2_ groups with the ones for the liposome. This spectral effect was indicative of the decrease in motional freedom of alkyl chains (increased order parameter). Moreover, insertion of the PSs into the membranes of the ML liposomes led to a shift to lower frequency in the CH bands and a lower frequency and broadening of the (PO^2−^) asymmetric stretching mode for the spectrum of phospholipid/cholesterol liposomes. This shows that a higher degree of hydration level in the head group region and a more ordered structure in the hydrocarbon tail region of ML liposomes occurred with the insertion of sterols (Figure 2d,e). On the other hand, considerable shift to the lower transmittance of amino +NH, hydroxyl–OH and phosphate PO^2−^ bands in the absence of sterols (Figure 2b) was indicative of a lower degree of hydration of the head group. Generally, the addition of sterols into the ML membrane system caused a narrowing in the bandwidth, which may indicate the immobilization of the phosphate groups. The pure sterols spectrum displayed weak broadband at 3180–3450 cm^−1^ that corresponds to OH stretch [69] (Figure 2d,e).

### 3.6. Stability of the Optimal Liposomes against Heat Treatment

**Figure 3:**
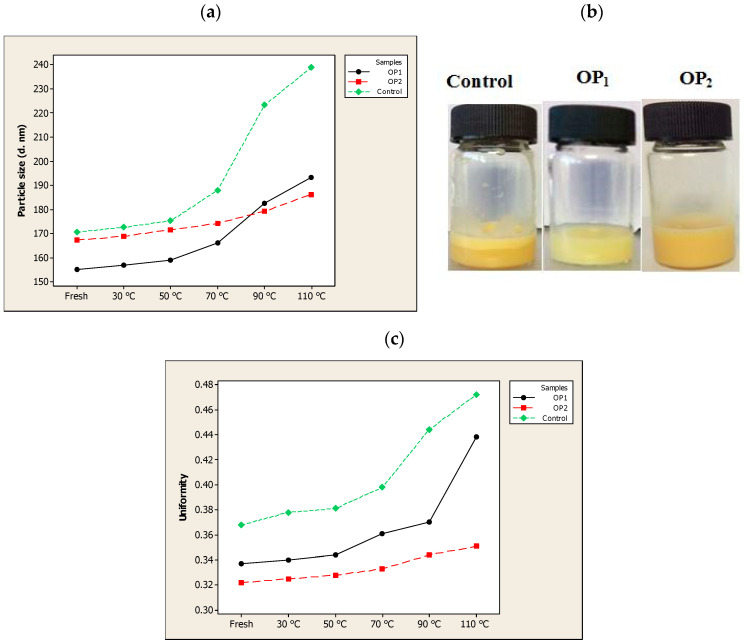
The effect of temperature (30–110 °C) on the (**a**) particle size, (**b**) appearance (at 90 °C) and (**c**) uniformity of optimal pre-liposome (OP_1_), optimal quercetin (QU)-loaded mixed soy lecithin (ML)-based liposome stabilized by β-sitosterol (βS) and stigmasterol (ST) (OP_2_) and empty mixed soy lecithin (ML)-based liposome (Control).

To continue the investigation of the stability in terms of particle size and uniformity in different composite liposomes, their thermal stability was studied from 30 to 110 °C for 30 min (Figure 3a–c). When the three samples (the optimal QU-loaded ML-based liposome stabilized by βS and ST (OP_2_), OP_1_ and empty liposome (Control)) were heat treated, the uniformity remained relatively constant up to 50 °C. The results also showed a slight but insignificant size enlargement as compared with the initial measurements (159 for OP_1_, 171.66 for OP_2_ and 175.33 nm for the control vesicles) upon heating the ML-based liposomes to 50 °C. These results suggest that the produced liposomes were relatively stable to thermal treatment at 50 °C for 30 min.

After heating at 70 °C, the non-nanoencapsulated compound vesicles (the control) experienced a significant (*p* < 0.05) increase in the particle size as well as the uniformity, and was almost decomposed and separated into two phases when heated at 90 °C (Figure 3a). It is considered that temperature, like light exposure, corresponds to energy input and able to impose changes in the crystalline structure of lipids, which lead to particle growth and subsequent gelling of lipid nanosystems [36]. When the temperature increases, there is a transition from the rigid gel phase of the membrane lipids to the liquid phase. As such, the mobility of the nanoparticles in the membrane increases rapidly which disturbs the lipid ordering, induces the phase transition leading to decreased anisotropy values and increased membrane fluidity. The destabilization process as a result of high temperatures also lead to a reduction in electrostatic repulsive forces and hence a decrease in the ZP. As reported by Freitas and Müller [70], rapid particle size growth was observed when stored at 50 °C, and associated with a decrease in their ZP.

During thermal treatments, OP_2_ exhibited the highest thermal stability; as it was fairly stable when treated up till 90 °C while the sample OP_1_ showed some particle aggregation and precipitation with a significant (*p* < 0.05) particle size increase when it was heated to 70 °C. A substantial reduction in the thermal stability of OP_1_ might be explained by the sticky phospholipid-squalene (squalene has an oily natural fraction) that sedimented after being heated at 70 °C for 30 min. However, it still showed no phase separation and displayed a relatively high uniformity. By having the highest negative ZP, OP_2_ was stable even after being heated at 90 °C, perhaps because electrostatic repulsive forces prevented it from fusing and aggregating. A high stability of OP_2_ composition at 90 °C heat for 30 min could also come from the steric stabilization by incorporating the βS and ST to the membrane that prevented the rapid release of the vesicular contents.

After heating above 100 °C in a water bath, major changes were observed in both particle size and uniformity of the composites OP_1_ and OP_2_. The diameter of OP_1_ and OP_2_ increased to 193.33 nm and 186.33 nm, respectively, after heating at 110 °C for 30 min. These results might be explained by two reasons. Firstly, high temperatures above 100 °C might lead to increased free fatty acid contents as a result of degradation of the phospholipids and acceleration of the hydrolysis of lecithin in the aqueous liposome dispersion [71]. Such chemical reactions can rupture the chemical bonds, therefore changing the permeability and electric potential of less rigid membranes (like the ML-based liposomes in this study), but these might be inhibited to some extent by adding membrane stabilizers like PSs. Secondly, the low cloud point (93 °C) of Tween 80 could lead to the breaking of hydrogen bonds between polyoxyethylene chain and water molecule followed by the significant decline of solubility, thus destroying the structure of liposome dispersions and resulting in phase separation.

### 3.7. Morphological Property

**Figure 4:**
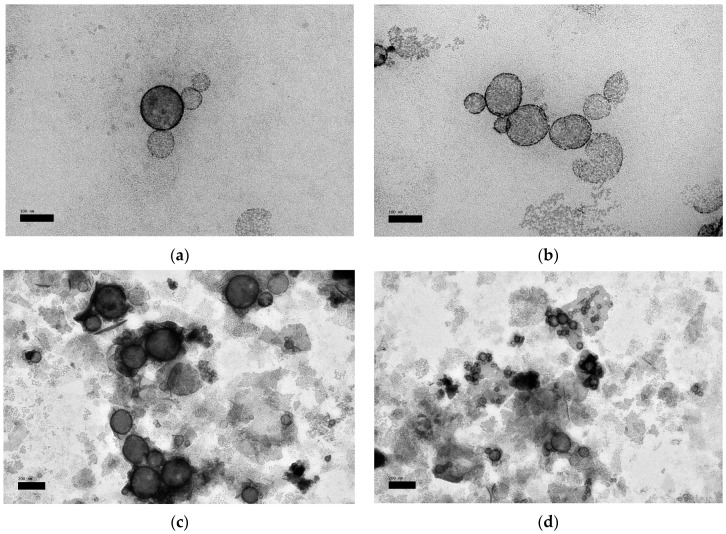

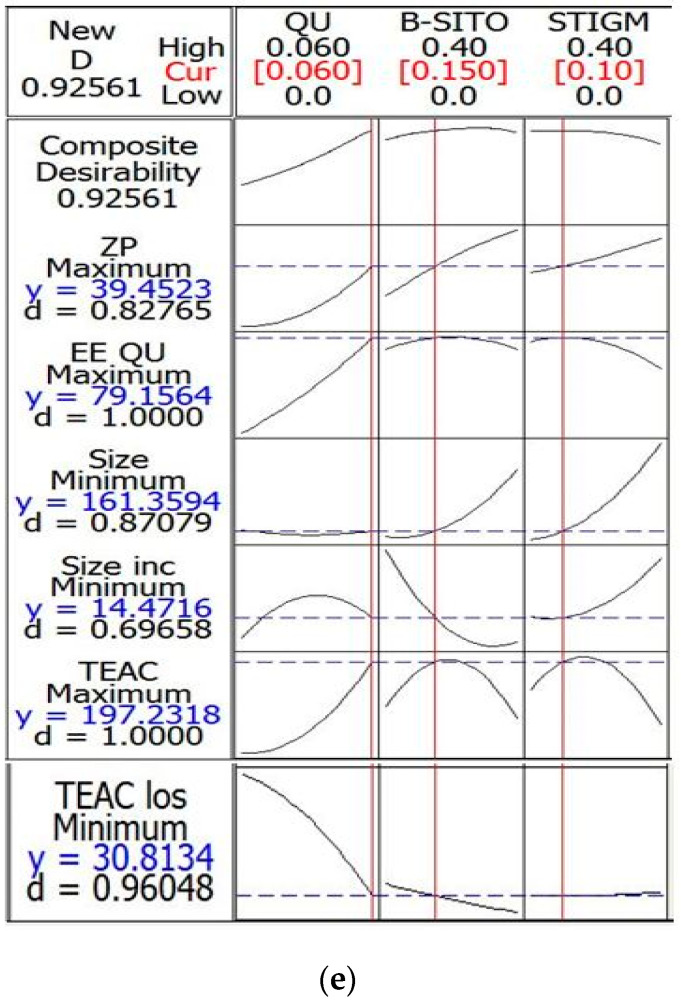
Transmission electron microscopy (TEM) micrographs of liposomes; (**a**) optimal quercetin (QU)-loaded mixed soy lecithin (ML)-based liposome stabilized by β-sitosterol (βS) and stigmasterol (ST) (OP_2_, scale bar: 100 nm), (**b**) optimal pre-liposome (OP_1_, scale bar: 100 nm), (**c**) optimal ultraviolet (UV)-treated quercetin (QU)-loaded mixed soy lecithin (ML)-based liposome stabilized by β-sitosterol (βS) and stigmasterol (ST) (OP_2_, scale bar: 200 nm), (**d**) optimal ultraviolet (UV)-treated pre-liposome (OP_1_, scale bar: 200 nm) and (**e**) multiple response optimizer showing the optimum concentrations of quercetin (QU), β-sitosterol (βS) and stigmasterol (ST) in order to achieve the maximum zeta potential (ZP), maximum quercetin (QU) encapsulation efficiency (EE_QU_), maximum Trolox equivalent antioxidant capacity (TEAC), minimum particle size, minimum %size increase and minimum loss of %Trolox equivalent antioxidant capacity (%TEAC).

TEM images of OP_2_ and OP_1_ (as a control) before and after UV light and the multiple response optimizer (Table 7) are illustrated in Figure 4a–e, respectively. As showed in Figure 4a, the TEM image displayed non-agglomerated liposomes with a mean particle size of 100–200 nm, and were spherical in shape. Compared to the control (Figure 4b), the morphology of the liposome in the present study revealed an enhanced rounded shape and smoothness. Images of UV-treated liposomes (Figure 4c,d) revealed aggregated vesicles and unfolded structures due to the damaging effect of photon energy on the membrane bilayers, which was much more notable in the control sample.

## 4. Conclusions

In this research, an extrusion method was successfully used to produce ML-based liposomes encapsulating QU with added mixture of PSs (βS and ST). The results showed that composition of the membrane critically affected the liposomal characteristics, whereby varying the concentrations of QU, βS and ST produced significant differences in terms of the ZP, QU entrapment efficacy and the photodegradation values of the liposomes. In summary, the colloidal and physical stabilities and antioxidative properties of the ML-based liposomes were governed primarily by the state of the active components embedded in the membranes. When QU was loaded in an appropriate amount, DPPH scavenging activity and the photostability of the liposomes were significantly improved. With the addition of βS and ST, a modification of acyl-chain order in liposome bilayers was observed, and this improved the liposomes stability against photo and thermal decomposition. The study also demonstrated that the designed ML liposomes with an inclusion of a helper lipid (squalene) and a non-ionic surfactant (Tween 80) could not tolerate a further intake of βS and ST, either individually or in a mixture. Additionally, ST was not as effective as βS at improving the vesicular integrity of the liposomes. The optimization procedure indicated that a stable liposome produced using 0.15 and 0.1 g/100 mL of βS and ST, respectively, could effectively encapsulate 0.06 g/100 mL of QU. This means that a combination of the selected PSs were able to improve QU entrapment efficacy when present at a suitable concentration in the ML membrane.

## Figures and Tables

**Table 1:** Range of components of mixed soy lecithin (ML).

Composition	Ingredients (%)
Phosphatidylcholine	19–21
Phosphatidylethanolamine	8–20
Inositol phosphatides	20–21
Other phosphatides	5–11
Soybean oil	33–35
Carbohydrates, free	2–5
Moisture	1

**Table 2:** Formulation compositions of liposomes; matrix of Box-Behnken Design (BBD).

Formulation Code	Runs	Quercetin, QU (X_1_, % *w*/*w*)	β-Sitosterol, βS (X_2_, % *w*/*w*)	Stigmasterol, ST (X_3_, % *w*/*w*)
F1	1	0	0.2	0.4
F2	2	0	0.2	0
F3	3	0.06	0.2	0.4
F4	4	0.03	0.4	0.4
F5	5	0	0.4	0.2
F6	6	0.06	0.2	0
F7	7 *	0.03	0.2	0.2
F8	8	0.06	0.4	0.2
F9	9	0.03	0.4	0
F10	10 *	0.03	0.2	0.2
F11	11	0	0	0.2
F12	12 *	0.03	0.2	0.2
F13	13	0.03	0	0.4
F14	14	0.06	0	0.2
F15	15	0.03	0	0

* Centre points.

**Table 3:** Changes in particle size and Trolox equivalent antioxidant capacity (TEAC) of the prepared liposomes before and after exposure to ultraviolet (UV) light (280–320 nm) for 6 h.

Formulation Code	Particle Size, d (nm)		TEAC (µM)	
-	Before	After	Before	After
F1	178 ± 1.45	211 ± 1.71	119.28	63.56
F2	158 ± 2.36	177 ± 5.36	130.41	73.01
F3	174 ± 1.78	204 ± 6.84	166.7	110.33
F4	184 ± 4.12	221 ± 9.27	70.17	33.14
F5	175 ± 6.19	202 ± 1.65	110.4	60.42
F6	161 ± 3.24	182 ± 5.14	177.2	123.35
F7	164 ± 8.17	189 ± 3.75	159.56	95.37
F8	171 ± 4.67	197 ± 6.54	171.12	115.88
F9	165 ± 3.39	188 ± 9.12	116.48	78.04
F10	165 ± 7.51	190 ± 3.20	162.99	96.83
F11	164 ± 4.84	190 ± 9.41	129.45	67.92
F12	166 ± 9.22	193 ± 1.43	159.8	93.55
F13	169 ± 7.43	197 ± 4.30	110.9	72.82
F14	161 ± 5.36	187 ± 7.83	174.95	122.61
F15	160 ± 4.55	189 ± 1.51	131.61	72.87

Values are the means ± SEM of two independent experiments each performed in triplicate.

**Table 4:** The particle size increase (%), poly dispersity index (PDI), Trolox equivalent antioxidant capacity (TEAC) loss (%), zeta potential (ZP) and the quercetin (QU) encapsulation efficiency (EE_QU_) (%) of the prepared liposomes.

Formulation Code	PDI	ZP (mV)	Particle Size Increase (%)	TEAC Loss (%)	EE_QU_ (%)
F1	0.180 ± 0.0049	−27.94 ± 1.33	18.53	46.71	-
F2	0.177 ± 0.0028	−33.33 ± 0.81	12.02	44.01	-
F3	0.192 ± 0.0035	−42.47 ± 2.51	17.24	33.81	65.74 ± 1.82
F4	0.253 ± 0.0063	−30.35 ± 1.32	20.1	52.77	33.41 ± 2.19
F5	0.199 ± 0.0063	−23.28 ± 2.63	15.42	45.27	-
F6	0.177 ± 0.0035	−39.76 ± 0.67	13.04	30.38	77.13 ± 2.84
F7	0.181 ± 0.0028	−33.49 ± 1.81	15.24	40.22	50.76 ± 0. 67
F8	0.188 ± 0.0056	−42.82 ± 0.41	15.2	32.28	69.59 ± 3.20
F9	0.176 ± 0.0042	−36.35 ± 0.94	13.93	33	47.76 ± 0.91
F10	0.207 ± 0.0035	−33.36 ± 2.16	15.15	40.59	52.19 ± 1.39
F11	0.211 ± 0.0063	−34.05 ± 1.39	15.85	47.53	-
F12	0.228 ± 0.0091	−33.71 ± 0.80	16.26	41.45	51.86 ± 3.44
F13	0.194 ± 0.0035	−35.29 ± 2.18	16.56	34.33	30.22 ± 0.49
F14	0.219 ± 0.0084	−39.05 ± 0. 73	16.14	29.91	73.19 ± 2.87
F15	0.230 ± 0.0028	−30.88 ± 1.39	18.12	44.63	42.51 ± 3.17

Values are the means ± SEM of two independent experiments each performed in triplicate.

**Table 5:** Analysis of variance for the experimental variables as linear, quadratic and interaction terms of each response variable (particle size, zeta potential (ZP), quercetin (QU) encapsulation efficiency (EE_QU_) and Trolox equivalent antioxidant capacity (TEAC)) and corresponding coefficients fitted for predictive models.

Source	Particle Size (nm)			ZP (mV)			EE_QU_ (%)			TEAC (µM)		
	Coefficient	F Ratio	*p* Value	Coefficient	F Ratio	*p* Value	Coefficient	F Ratio	*p* Value	Coefficient	F Ratio	*p* Value
Model	-	-	-	-	-	-	-	-	-	-	-	-
Linear	-	-	-	-	-	-	-	-	-	-	-	-
b1	−0.000	0.00	1.000 ^c^	−130.33	5.03	0.075 ^c^	586.82	28.79	0.003 ^a^	−190.4	0.23	0.653 ^c^
b2	−8.125	0.79	0.414 ^c^	−2.32	0.07	0.801 ^c^	69.34	17.86	0.008	240.8	16.21	0.010
b3	11.875	1.69	0.250 ^c^	−3.64	0.17	0.693 ^c^	49.79	9.21	0.029	233.7	15.26	0.011
Quadratic	-	-	-	-	-	-	-	-	-	-	-	-
b11	555.556	0.47	0.522 ^c^	2187.50	8.04	0.036 ^b^	3880.09	7.15	0.044 ^c^	14891.2	7.92	0.037 ^b^
b22	56.250	9.59	0.027 ^b^	−17.22	0.98	0.367 ^c^	−151.70	21.57	0.006	−692.6	33.83	0.002 ^a^
b33	56.250	9.59	0.027 ^b^	9.66	0.31	0.602 ^c^	−176.51	29.21	0.003 ^a^	−644.7	29.31	0.003
Interaction	-	-	-	-	-	-	-	-	-	-	-	-
b12	−41.667	0.13	0.735 ^c^	605.83	29.69	0.003 ^a^	−117.92	0.32	0.597 ^c^	634.2	0.69	0.444 ^c^
b13	−291.667	6.28	0.054 ^c^	337.50	9.21	0.029	−166.67	0.63	0.462 ^c^	26.2	0.00	0.974 ^c^
b23	62.500	12.82	0.016 ^a^	−65.06	15.22	0.011	−12.88	0.17	0.699 ^c^	−160.0	1.96	0.221 ^c^
Lack of fit	-	2.58	0.291 ^c^	-	94.12	0.011	-	18.07	0.053 ^c^	-	37.41	0.026
R^2^	98.73%	-	-	97.71%	-	-	99.35%	-	-	96.88%	-	-
Adj-R^2^	96.43%	-	-	93.59%	-	-	98.18%	-	-	91.27%	-	-
precision	-	-	-	-	-	-	-	-	-	-	-	-

bi represents the estimated regression coefficient for the main linear effects. bii represents the estimated regression coefficient for the quadratic effects, and bij represents the estimated regression coefficient for the interaction effect 1: quercetin (QU) concentration (X1); 2: β-sitosterol (βS) concentration (X2); 3: stigmasterol (ST) concentration (X3). Significant at *p* < 0.05; ^a^ the most significant (*p* < 0.05); ^b^ the least significant (*p* < 0.05); ^c^ nonsignificant (*p* > 0.05).

**Table 6:** Analysis of variance for the experimental variables as linear, quadratic and interaction terms of each response variable (poly dispersity index (PDI), particle size increase and Trolox equivalent antioxidant capacity (TEAC) loss (%)) and corresponding coefficients fitted for predictive models.

Source	PDI			Particle Size Increase (%)			TEAC Loss (%)		
	Coefficient	F Ratio	*p* Value	Coefficient	F Ratio	*p* Value	Coefficient	F Ratio	*p* Value
Model	-	-	-	-	-	-	-	-	-
Linear	-	-	-	-	-	-	-	-	-
b1	1.1903	2.50	0.174 ^c^	84.92	3.46	0.122 ^c^	−134.06	4.79	0.080 ^c^
b2	−0.2946	6.81	0.048 ^b^	−20.65	9.09	0.030 ^b^	−41.30	20.20	0.006
b3	−0.0452	0.16	0.705 ^c^	−3.11	0.21	0.669^c^	−30.80	11.23	0.020
Quadratic	-	-	-	-	-	-	-	-	-
b11	−18.2407	3.34	0.127 ^c^	−1037.50	2.93	0.148 ^c^	−2478.24	9.29	0.028 ^b^
b22	0.3833	2.91	0.149 ^c^	25.91	3.61	0.116 ^c^	5.61	0.09	0.771 ^c^
b33	−0.1854	0.68	0.447 ^c^	14.78	1.18	0.328 ^c^	5.11	0.08	0.791 ^c^
Interaction	-	-	-	-	-	-	-	-	-
b12	−0.7917	0.30	0.606 ^c^	−21.25	0.06	0.817 ^c^	192.92	2.71	0.161 ^c^
b13	0.5000	0.12	0.742 ^c^	−96.25	1.21	0.321	30.42	0.07	0.806 ^c^
b23	0.7063	10.71	0.022 ^a^	48.31	13.60	0.014 ^a^	187.94	15.22	0.000 ^a^
Lack of fit	-	0.23	0.870 ^c^	-	4.15	0.200 ^c^	-	7.61	0.118 ^c^
R^2^	80.52%	-	-	90.96%	-	-	98.60%	-	-
Adj−R^2^	45.46%	-	-	74.69%	-	-	96.08%	-	-
CV	-	-	-	-	-	-	-	-	-
Adequate	-	-	-	-	-	-	-	-	-
precision	-	-	-	-	-	-	-	-	-

bi represents the estimated regression coefficient for the main linear effects. bii represents the estimated regression coefficient for the quadratic effects, and bij represents the estimated regression coefficient for the interaction effect 1: quercetin (QU) concentration (X1); 2: β−sitosterol (βS) concentration (X2); 3: stigmasterol (ST) concentration (X3). Significant at *p* < 0.05; ^a^ the most significant (*p* < 0.05); ^b^ the least significant (*p* < 0.05); ^c^ nonsignificant (*p* > 0.05).

**Table 7:** The predicted and experimental values of the response variables of optimized liposome (0.06% (*w*/*w*) quercetin (QU), 0.15% (*w*/*w*) β-sitosterol (βS) and 0.1% (*w*/*w*) stigmasterol (ST)).

Response Variables	Experimental Value	Predicted Value	Desirability
Particle size (nm) ± SD	161.69 ± 1.98	161.35	0.870
ZP (mV) EE (%)	−37.85 ± 0.48 79.36 ± 2.93	−39.45 79.15	0.827 1
TEAC (µM)	198.00 ± 3.40	197.23	1
Particle size increase (%)	16.28 ± 1.03	14.47	0.696
TEAC loss (%)	32.54 ± 1.93	30.81	0.960
Composite			0.925

Values are the means ± SEM of two independent experiments each performed in triplicate. ZP, EE and TEAC are zeta potential, encapsulation efficiency and trolox equivalent antioxidant capacity, respectively.
